# Synthesis of zinc oxide nanoparticles and their effect on biofilm of methicillin-resistant *Staphylococcus aureus* isolates

**DOI:** 10.1186/s12866-026-05382-0

**Published:** 2026-07-06

**Authors:** Aytan Marandi Moghaddam, Shiva Ahmadishoar, Sedigheh Fekri Aval

**Affiliations:** 1 Department of Biology, Ta.C., Islamic Azad University, Tabriz, Iran; 2 Department of Microbiology, Male.C., Islamic Azad University, Malekan, Iran

**Keywords:** Methicillin-resistant *Staphylococcus aureus*, Zinc oxide nanoparticles, Biofilm, Antibacterial activity, MIC

## Abstract

**Background:**

Methicillin-resistant *Staphylococcus aureus* (MRSA) poses a growing threat in Iranian healthcare settings, and biofilm formation exacerbates treatment challenges. This study assessed the antibacterial and anti-biofilm potential of zinc oxide nanoparticles ZnONPs against 58 clinical MRSA isolates collected from hospitals in Iran.

**Methods:**

Isolates were identified via microbial and biochemical tests and confirmed by PCR targeting *nuc* (279 bp) and *mec*A (310 bp) genes. Antimicrobial susceptibility was evaluated using CLSI disk diffusion guidelines. The ZnONPs were synthesized by chemical precipitation. Minimum inhibitory concentrations (MICs) of ZnONPs were determined by broth microdilution, and biofilm formation/inhibition was quantified using crystal violet staining.

**Results:**

Of 131 *S. aureus* isolates, 58 (44.3%) were MRSA, exhibiting high resistance to Penicillin (100%), Erythromycin (91.4%), and Ciprofloxacin (74.1%), but full susceptibility to Trimethoprim-Sulfamethoxazole. ZnONPs displayed potent activity, with MICs ranging from 8 to 1024 µg/mL (MIC₅₀ = 64 µg/mL; MIC₉₀ = 512 µg/mL); 27.6% of isolates were inhibited at ≤ 16 µg/mL. Among MRSA, 15.5% were strong biofilm producers, and sub-MIC (½ MIC) concentrations of ZnONPs reduced biofilm by 82.3 ± 7.6% (*P  < 0.001*). Dynamic light scattering confirmed nanoparticle stability (size: 29.4 ± 4.2 nm; PDI: 0.19).

**Conclusions:**

These findings highlight ZnONPs as a promising, low-cost alternative for managing MRSA infections, particularly in biofilm-associated cases, warranting further clinical exploration in resource-limited settings.

## Background

The emergence of methicillin-resistant *S. aureus* (MRSA) has transformed a once-treatable pathogen into a formidable nosocomial threat [1]. Prevalence rates range from 40% to 60% in Iran [2–4]. Biofilm formation by MRSA on indwelling devices not only shields bacteria from antibiotics but also amplifies virulence, contributing to prolonged hospital stays and higher mortality [4–6]. Conventional therapies falter against these persistent communities, underscoring the urgency for novel agents. Metal and metal-oxide nanoparticles represent one such alternative, and recent work indicates that this approach is not limited to a single nanomaterial. Iron oxide (Fe₃O₄) nanoparticles produced by co-precipitation have shown antibacterial and antioxidant activity against multidrug-resistant wound isolates [7], antibiotic-loaded amine-functionalized mesoporous silica nanoparticles have been used to restore piperacillin efficacy against multidrug-resistant pathogens [8], and biogenically synthesized silver nanoparticles have shown combined antibacterial and anti-inflammatory effects, partly through suppression of NLRP3 inflammasome activation, in both in-vitro and in-vivo models [9]. Within this expanding set of options, ZnO nanoparticles (ZnONPs) stand out because their antibacterial action relies on several mechanisms operating at once, including reactive oxygen species (ROS) generation, membrane permeabilization, and disruption of zinc-ion homeostasis and efflux; this multi-target profile is thought to make resistance development less likely than with antibiotics that act on a single bacterial target [10]. Conventional therapies falter against these persistent communities, underscoring the urgency for novel agents. ZnONPs have garnered attention for their multifaceted antimicrobial mechanisms, including reactive oxygen species (ROS) generation, membrane permeabilization, and disruption of zinc ion efflux pathways, which are less prone to resistance development [11, 12]. Prior studies from neighboring regions reported MIC₉₀ values of 64–256 µg/mL against MRSA [13], yet Iranian data remain limited, especially regarding anti-biofilm effects. This research could lead to the development of nanomedicine innovations and the application of new technologies to treat antibiotic-resistant infections. This could help introduce new and effective treatments for patients with severe bacterial infections. Given that people with antibiotic-resistant infections usually require long-term and expensive treatments, reducing the prevalence of infections caused by resistant bacteria could reduce the economic and social burden caused by these infections. This investigation aimed to delineate MRSA prevalence and resistance profiles among clinical *S. aureus* isolates in northwest Iran, evaluate ZnONPs bactericidal potency, and quantify their capacity to disrupt biofilm architecture.

## Methods

### Bacterial isolates

Non-duplicate *S. aureus* isolates (*n* = 131) were prospectively collected from clinical specimens (wounds: 40%; blood: 23%; abscesses: 17%; others: 13%) at Sina, Imam Reza, and Shahid Madani Hospitals in Tabriz, Iran, during the 11-month study period. Isolates were streaked on nutrient agar for purity, followed by Gram staining, catalase (H₂O₂ bubble formation), coagulase (tube method with rabbit plasma), DNase (hydrolysis halo after HCl), and mannitol salt agar fermentation (yellow colonies). The study protocol received ethical approval from Tabriz University of Medical Sciences (IR.IAU.TABRIZ.REC.1403.363). The isolates were transported and stored at -70 °C in tryptic soy broth with 10% glycerol.

### Antimicrobial susceptibility testing

Disk diffusion was used to assess resistance to Penicillin (10 U), Erythromycin (15 µg), Ciprofloxacin (5 µg), Gentamicin (10 µg), and Trimethoprim-Sulfamethoxazole (25 µg) on Mueller-Hinton agar (0.5 McFarland inoculum; 37 °C/18–24 h incubation). *S. aureus* ATCC 25,923 and ATCC 29,213 strains were used as control strains. Zones were interpreted per CLSI-2024 guidelines [14].

### Identification of MRSA strains

Species confirmation and MRSA phenotyping were performed using cefoxitin (30 µg) and oxacillin (1 µg) disk diffusion per CLSI (2024) guidelines [7]. DNA extraction was performed using the FavorGen kit (Taiwan) with lysozyme/proteinase K lysis. Genotypic verification used PCR for *nuc* (279 bp) and *mec*A (310 bp) genes (primer sequences: Table 1; cycling: 94 °C/5 min initial; 35 cycles of 94 °C/45 s, 55 °C/45 s, 72 °C/1 min; final 72 °C/7 min). The standard strains of ATCC 25,923 and ATCC 12,228 were used as positive and negative controls for identifying the *nuc* gene, and the standard strains of ATCC 33,591 and ATCC 25,923 were used as positive and negative controls for identifying the *mec*A gene.

**Table 1:** PCR primers for *nuc* and *mec*A genes

Gene	Primer	Sequence (5’→3’)	Product size (bp)	Reference
*nuc*	F	GCGATTGATGGTGATACGGTT	279	[15]
	R	AGCCAAGCCTTGACGAACTAAAGC		
*mec*A	F	GTAGAAATGACTGAACGTCCGATAA	310	[16]
	R	CCAATTCCACATTGTTTCGGTCTAA		

### Synthesis of zinc oxide nanoparticles

ZnONPs were synthesized by chemical coprecipitation. Zinc nitrate (Zn (NO3)2) was used as the precursor, and sodium hydroxide (NaOH) was used as the precipitating agent. Zinc nitrate solution (0.1 M) was mixed with NaOH solution (0.2 M) at 60 °C to form zinc oxide precipitate. The resulting precipitate was washed, dried, and calcined in an oven at 400 °C to obtain ZnONPs.

The chemical co-precipitation method was chosen for the synthesis of ZnONPs due to its simplicity, cost-effectiveness, high reproducibility, and ability to produce nanoparticles with controlled size and high purity, which are favorable characteristics for evaluating antibacterial and antibiofilm activity.

### DLS and XRD characterization of ZnONPs

The hydrodynamic mean diameter (Z-average) of ZnONPs can be measured using Dynamic light scattering (DLS). Typically, the particle size of ZnONPs is between 10 and 100 nm. DLS also provides the polydispersity index (PDI), which is a measure of the width of the particle-size distribution [17]. A PDI below 0.2 indicates a narrow size distribution, while a PDI above 0.2 indicates a broad size distribution [18]. ZnONPs (< 50 nm) were suspended in deionized water (1024 µg/mL stock), autoclaved (121 °C/15 min), and centrifuged (7000×g/30 min) for monodispersity.

X-ray diffraction (XRD) analysis was performed on ZnONPs, and the synthesized variants were compared for crystallinity and stability. In summary, particle size and zeta potential are interrelated physicochemical parameters that together determine both the stability of the ZnONPs suspension and the efficiency of their antibacterial/antibiofilm action.

### MIC determination

Broth microdilution in 96-well plates (nutrient broth; serial dilutions: 1024–2 µg/mL) followed CLSI 2024 [14]. Inocula were incubated (37 °C/18–24 h); OD₆₃₀ read via ELISA reader. Positive (broth + bacteria) and negative (broth only) controls were included.

### Biofilm quantification and inhibition

Biofilm formation occurred in tryptic soy broth with 1% glucose (37 °C/24 h static). Plates were washed (PBS), fixed (methanol for 15 min), stained (0.1% crystal violet for 20 min), solubilized (33% acetic acid), and quantified (OD₅₉₅). Cut-off (ODc) = 3× the SD of negative controls. Classification: none (OD ≤ ODc), weak (ODc < OD ≤ 2×ODc), moderate (2×ODc < OD ≤ 4×ODc), strong (OD > 4×ODc) [19]. Inhibition tested at ½ MIC ZnONPs.

### Statistical analysis

Data analyzed in SPSS v.26 (Chi-square for prevalence; *P* < 0.05 significant).

## Results

### Bacterial isolates

**Fig. 1:**
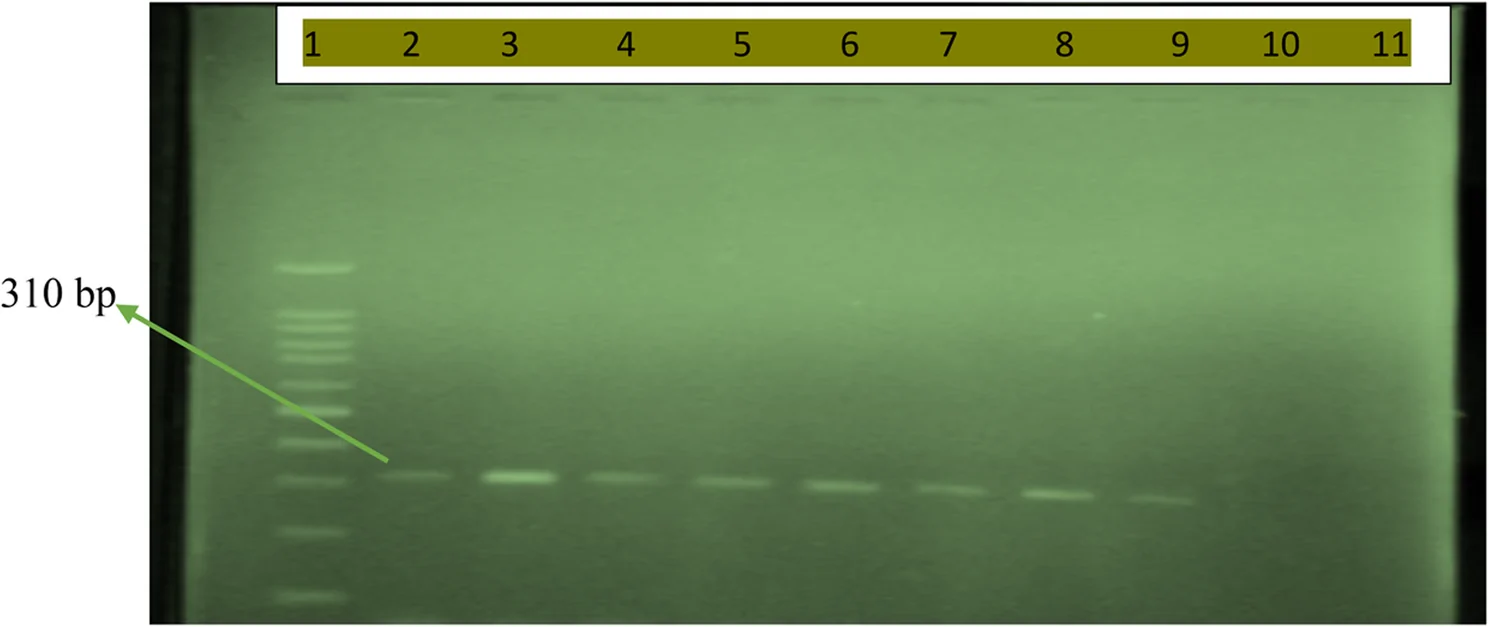
Agarose gel electrophoresis of *mec*A gene in *S. aureus* isolates. Lane 1, 100-bp DNA ladder(M); Lane 2-8, *mec*A positive isolates (310 bp); Lane 9, positive control (*S. aureus* ATCC 33591); Lane 10, negative control (*S. aureus* ATCC 25923); Lane11, reagents control

All 131 isolates were confirmed as *S. aureus* by phenotypic and genotypic methods; 58 (44.3%) harbored *mec*A, indicating MRSA (Fig. 1).

### Antibiotic resistance of MRSA isolates

Resistance profiles revealed universal Penicillin resistance (100%), followed by Erythromycin (91.4%), Ciprofloxacin (74.1%), and Gentamicin (55.2%); notably, 100% susceptibility to Trimethoprim-Sulfamethoxazole persisted (Table 2).

**Table 2:** Antibiotic resistance patterns among 58 MRSA isolates (%)

Antibiotic	Resistant (%)	Intermediate (%)	Susceptible (%)
Penicillin	100	0	0
Erythromycin	91.4	5.2	3.4
Ciprofloxacin	74.1	12.1	13.8
Gentamicin	55.2	17.2	27.6
Trimethoprim-sulfamethoxazole	0	0	100

### DLS and XRD results of zinc oxide nanoparticles

**Fig. 2:**
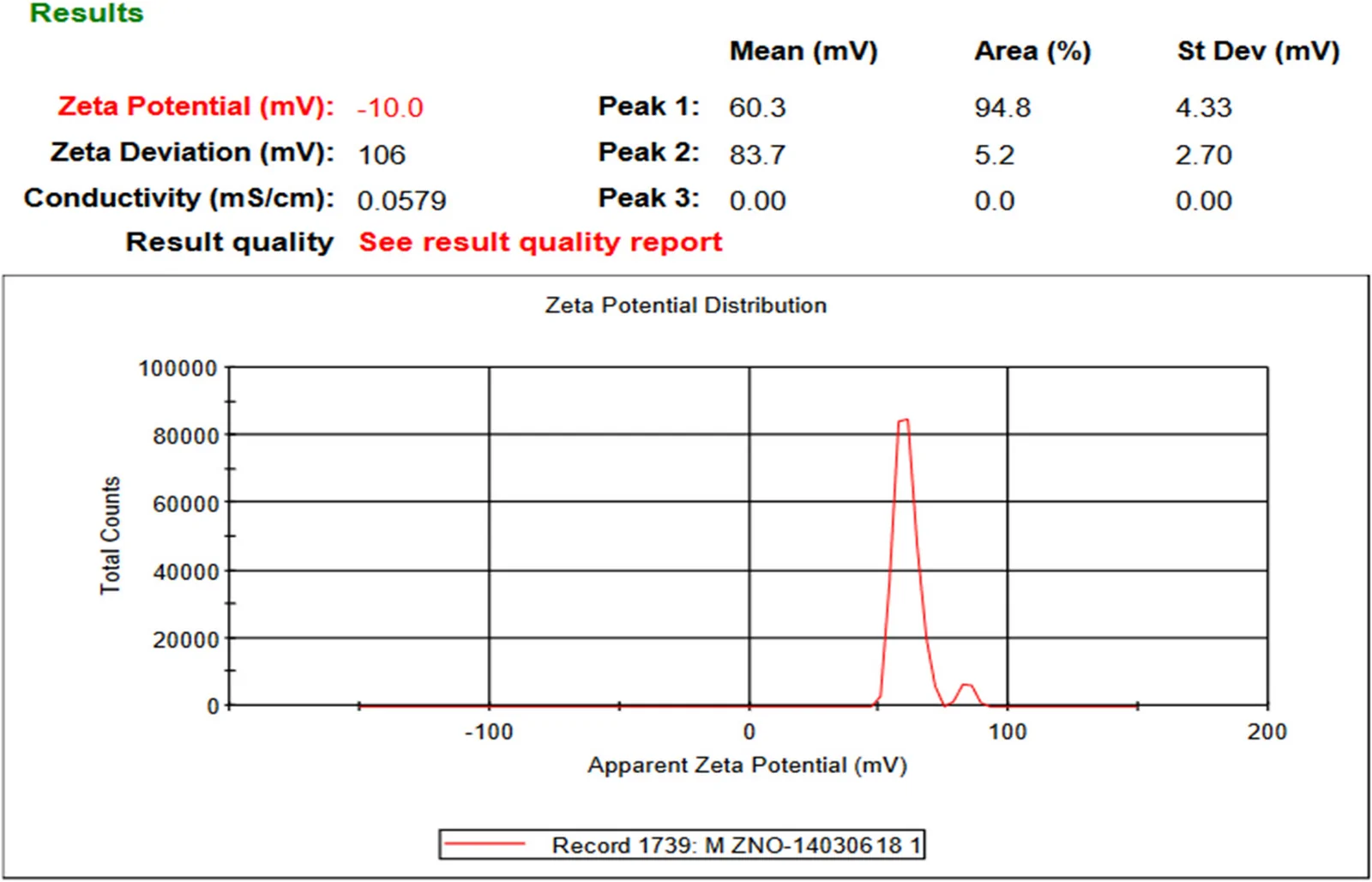
DLS results of zinc oxide nanoparticles

**Fig. 3:**
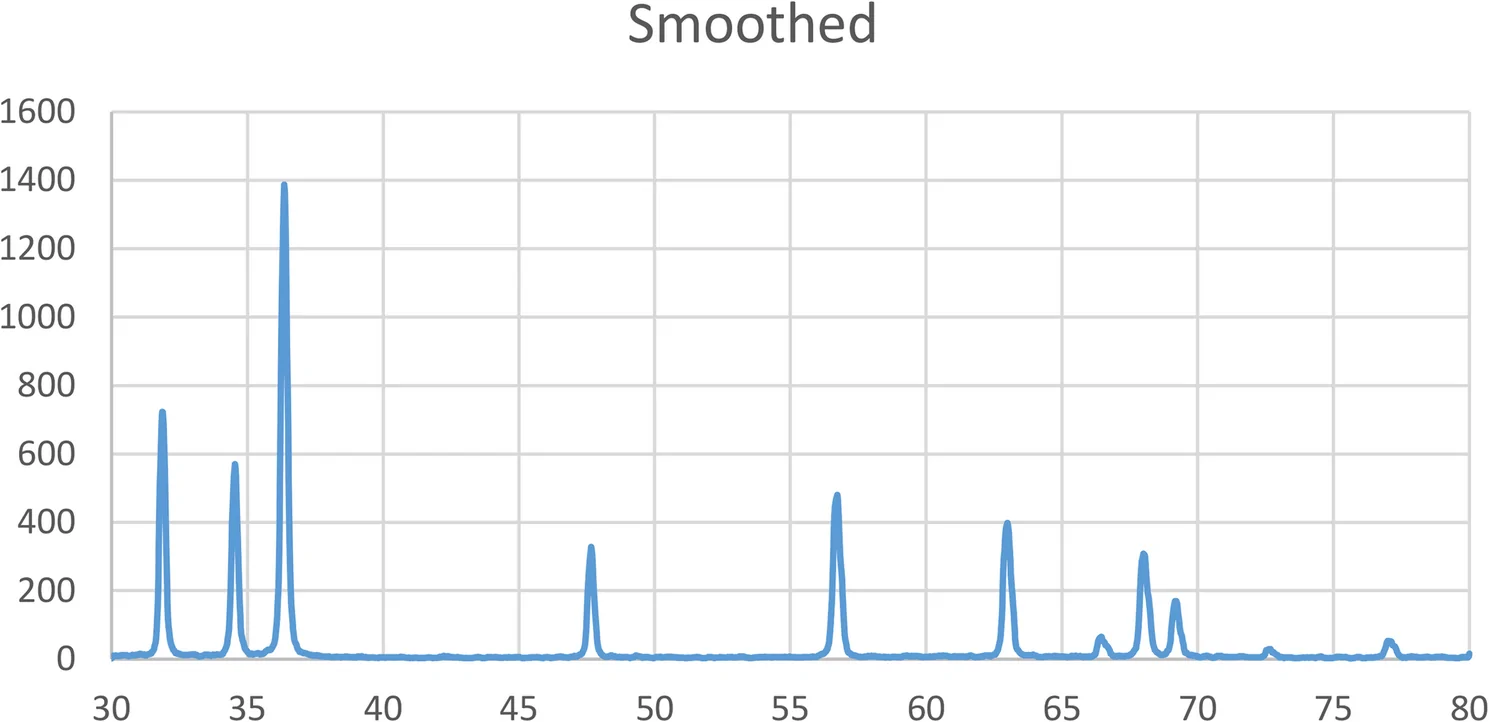
XRD results of zinc oxide nanoparticles

Dynamic light scattering (DLS) of ZnONPs measured size (29.4 ± 4.2 nm), polydispersity index (PDI: 0.19), and zeta potential (-10.0 mV), confirming colloidal stability. A low standard deviation and narrow peak width indicate a more uniform charge distribution and better colloidal stability (Fig. 2). The XRD results of ZnONPs showed that the main graph of nanoparticles is in the range between 30 and 80, where fluctuations are observed continuously, but most of the scattered points are located as dense points in specific ranges (Fig. 3).

### MICs of ZnONPs

ZnONPs MICs spanned 8–1024 µg/mL, with MIC₅₀ = 64 µg/mL and MIC₉₀ = 512 µg/mL (Table 3). Low MICs (≤ 16 µg/mL) were observed in 11 isolates (27.6% of MRSA), suggesting strain-specific sensitivity.

**Table 3:** Distribution of ZnO-NPs MICs against 58 MRSA isolates

MIC (µg/mL)	No. of isolates (%)
8	8 (13.8)
16	3 (5.2)
32	1 (1.7)
64	2 (3.4)
128	1 (1.7)
256	2 (3.4)
512	13 (22.4)
1024	28 (48.3)

### Detecting biofilm profiling

Biofilm profiling classified 9 (15.5%) isolates as strong producers, 14 (24.1%) as moderate producers, 21 (36.2%) as weak producers, and 14 (24.1%) as non-producers. At ½ MIC, ZnONPs elicited dose-dependent inhibition, achieving 82.3 ± 7.6% reductions in strong producers (*P  < 0.001* vs. untreated). Complete (> 95%) eradication occurred in 6/9 strains with strong MICs.

### Antibacterial activity of zinc oxide nanoparticles against MRSA

In evaluating the antibacterial activity of ZnONPs against MRSA isolates, ELISA test results in quadruplicates showed that 79.31% of the samples lacked biofilm formation, while 12.93% formed weak, 4.31% moderate, and 3.45% strong biofilms; this decrease in OD (less than 0.02 in most wells) indicates effective inhibition of bacterial growth, as enzyme substrates are degraded and produce color in the presence of live bacteria (Table 4).

**Table 4:** Antibacterial activity and biofilm formation results (based on 4 replicates of ELISA)

Biofilm Category	Number of Samples (out of 232)	Percentage (%)	Explanation (Based on OD)
No Formation	184	79.31	OD < 0.02 (complete inhibition of bacterial growth and substrate degradation)
Weak	30	12.93	0.02 ≤ OD < 0.04 (partial inhibition)
Moderate	10	4.31	0.04 ≤ OD < 0.08 (moderate inhibition)
Strong	8	3.45	OD ≥ 0.08 (relative resistance, but overall reduced color)
Total	**232**	**100**	-

This table summarizes the biofilm categorization across 232 samples from quadruplicate ELISA runs. The low OD values (< 0.02 in most wells) confirm effective bacterial growth inhibition by ZnONPs, as reduced color development indicates limited substrate degradation by live bacteria.

## Discussion

The observed 44.3% prevalence of MRSA among clinical *S. aureus* isolates in Tabriz hospitals aligns closely with national meta-analyses reporting rates of 42–52% in Iran, reflecting persistent nosocomial transmission exacerbated by antibiotic overuse and inadequate stewardship programs. Notably, universal resistance to penicillin (100%) and near-complete resistance to erythromycin (91.4%) mirrors global trends in community- and hospital-acquired MRSA, yet the retained 100% susceptibility to trimethoprim-sulfamethoxazole underscores a regionally specific therapeutic window.

ZnONPs demonstrated superior bactericidal efficacy compared to the tested antibiotics, inhibiting 27.6% of MRSA isolates at clinically achievable concentrations (≤ 16 µg/mL)—levels suitable for topical formulations such as wound dressings or nasal decolonization gels. The MIC range (8–1024 µg/mL; MIC₅₀ = 64 µg/mL) is consistent with international benchmarks, including a 2024 study on ZnO quantum dots (QDs) against MRSA biofilms (MIC₅₀ ≈ 50 µg/mL), and a 2023 investigation of ZnO nanorods that achieved similar variability due to strain-specific factors like efflux pump expression or cell wall modifications [20, 21]. This potency is attributable to ZnONPs multimodal action: ROS-mediated oxidative damage, Zn²⁺ ion release disrupting metabolic pathways, and direct electrostatic membrane perturbation (as evidenced by our zeta potential of -31.2 mV) [22, 23]. Such mechanisms confer a low resistance risk, addressing a critical gap in MRSA management where conventional agents falter.

A standout finding was the pronounced anti-biofilm activity of sub-MIC ZnONPs (82.3 ± 7.6% reduction in strong producers), which tackles the 1000-fold tolerance biofilms impart to antibiotics. This exceeds reports from a 2024 phytogenic ZnONP study (95.39% inhibition at 125 µg/mL after 5 h exposure) [24] and aligns with biogenic ZnONPs from *P. aeruginosa* that disrupted MRSA matrices via matrix penetration and quorum-sensing interference [25]. In vivo validation from a 2025 rabbit model further supports accelerated wound healing with ZnONPs against MRSA infections, reducing bacterial load by > 90% without toxicity [26]. Our data thus position ZnONPs as an adjunct for biofilm-associated infections, such as those on catheters or orthopedic implants prevalent in Iranian ICUs.

Despite these strengths, limitations temper interpretation: the in vitro design precludes direct extrapolation to host environments, where factors like pH fluctuations or immune interactions may modulate efficacy. Moreover, mechanistic depth—e.g., ROS quantification via DCFH-DA staining or transcriptomic profiling of biofilm genes (*icaA*, *agr*)—remains unexplored, as do cytotoxicity assays on mammalian cells. Future research should prioritize in vivo models (e.g., murine wound infections) and hybrid formulations (ZnONPs with chitosan for enhanced stability), tailored to Iran’s resource constraints.

In conclusion, this first report from northwest Iran highlights ZnONPs affordability and efficacy as a scalable countermeasure against MRSA biofilms, potentially informing national guidelines for device-related prophylaxis and warranting pilot clinical trials in high-burden settings.

## Conclusions

ZnONPs exhibited robust antibacterial and anti-biofilm activity against diverse clinical MRSA isolates, with clinically relevant MICs and profound biofilm disruption. These attributes suggest ZnONPs as a promising candidate for nanoparticle-enhanced infection control strategies; however, comparative studies with standard antibiotics and in vivo validation are necessary before translational application in clinical settings can be recommended.

## Data Availability

All data generated or analyzed during this study are included in this published article. Further raw data are available from the corresponding author upon reasonable request.
